# Prediagnostic Urinary Tract-Related Manifestations and Recurrence Risk in Colorectal Cancer with Bladder Involvement

**DOI:** 10.3390/jcm15155867

**Published:** 2026-07-27

**Authors:** Yi-Huei Chang, Yi-Chang Chen, Wen-Chi Chen, Tao-Wei Ke, Chi-Ping Huang, Laing-You Wu, Hui-Tsung Hsu, Chi-Jung Chung

**Affiliations:** 1 Department of Urology, China Medical University Hospital, Taichung 40447, Taiwan; 021959@tool.caaumed.org.tw (Y.-H.C.);; 2 Department of Public Health, College of Public Health, China Medical University, No. 100, Sec. 1, Jingmao Rd., Beitun Dist., Taichung 406040, Taiwan; 3 School of Medicine, College of Medicine, China Medical University, Taichung 406040, Taiwan; 4 Department of Colorectal Surgery, China Medical University Hospital, Taichung 40447, Taiwan; 5 Department of Medical Research, China Medical University Hospital, Taichung 40447, Taiwan

**Keywords:** colorectal cancer, bladder involvement, urinary tract-related manifestations, inverse probability of treatment weighting, retrospective cohort study

## Abstract

**Background/Objectives:** Colorectal cancer (CRC) with bladder involvement represents a distinct subgroup of locally advanced disease frequently accompanied by urinary tract-related manifestations (UTRMs). However, the clinical relevance of prediagnostic UTRMs in this high-risk population remains unclear. This study investigated the association between UTRMs and oncologic outcomes in patients with bladder-involved CRC. **Methods:** We conducted a population-based retrospective cohort study using the National Health Insurance Research Database and Catastrophic Illnesses Database (2000–2020). A total of 2608 patients with CRC involving the bladder were included. Patients with prediagnostic UTRMs, including urinary frequency, urgency, dysuria, hematuria, and urinary tract infections, were identified using diagnostic codes recorded within 6 months before CRC diagnosis. Inverse probability of treatment weighting was applied to balance baseline characteristics. Patients were followed from CRC diagnosis until disease recurrence, death, or end of the study period. Weighted Cox proportional hazards models were used to estimate hazard ratios (HRs) and 95% confidence intervals (CIs). **Results:** Patients with UTRMs had a significantly higher risk of disease recurrence than those without UTRMs (adjusted HR 1.57). Patients with UTRMs requiring antibiotic treatment exhibited an even greater recurrence risk (adjusted HR 4.17), although this finding should be interpreted cautiously because of the limited sample size. Associations between UTRMs and recurrence were generally consistent across subgroup analyses. In contrast, UTRMs were not significantly associated with CRC-specific mortality. **Conclusions:** Prediagnostic UTRMs were associated with an increased risk of recurrence in patients with CRC involving the bladder. These readily obtainable preoperative clinical manifestations may provide supplementary information for postoperative surveillance in this high-risk population. Further studies are warranted to validate these findings.

## 1. Introduction

Colorectal cancer (CRC) remains a major global health burden, accounting for approximately 9.6% of all cancer diagnoses and 9.3% of cancer-related deaths worldwide [1]. Patients with CRC may present with rectal bleeding, altered bowel habits, anemia, or abdominal pain; however, a substantial proportion remain asymptomatic until disease reaches an advanced stage [1,2,3]. Approximately 10% of patients with CRC present with locally advanced disease involving the peritoneum (T4a) or adjacent organs (T4b) at diagnosis [4]. CRC involving adjacent organs such as the bladder is generally categorized as locally advanced (T4b) disease according to the American Joint Committee on Cancer staging system [5]. In particular, CRC with bladder involvement often requires extensive pelvic surgery and multidisciplinary management because of local tumor extension and complex pelvic anatomy. Despite aggressive surgical treatment, recurrence rates ranging from 10% to 36% have been reported in patients with CRC involving the bladder, highlighting the need for a better understanding of clinical factors associated with recurrence [6,7,8,9,10,11,12].

When CRC involves adjacent organs such as the bladder, patients may present with a spectrum of urinary tract-related manifestations (UTRMs), including urinary frequency, urgency, dysuria, hematuria, or urinary tract infections. Because UTRMs are generally related to involvement of adjacent urinary structures, they are not typically considered common presenting features of CRC and may suggest local disease extension when present [10,11]. Owing to their nonspecific nature, these manifestations often overlap with common benign lower urinary tract conditions encountered in primary care and urology practice [13,14,15]. Consequently, UTRMs may be overlooked or attributed to nonmalignant etiologies, potentially delaying recognition of the underlying disease.

The development of UTRMs in CRC is likely multifactorial. Local tumor invasion and associated inflammatory reactions may directly impair bladder function, disrupt adjacent tissues, and contribute to lower urinary tract dysfunction, resulting in urinary symptoms [9,10,11]. Given that UTRMs are readily identifiable clinical manifestations, they may also provide important prognostic information in patients with CRC involving the bladder. However, despite these plausible mechanisms, the association between prediagnostic UTRMs and oncologic outcomes in patients with CRC involving the bladder remains poorly defined.

Therefore, this study aimed to investigate whether prediagnostic UTRMs are associated with disease recurrence and CRC-specific mortality in patients with CRC involving the bladder using a nationwide population-based cohort.

## 2. Materials and Methods

### 2.1. Data Source

This population-based retrospective cohort study was conducted using nationwide healthcare databases in Taiwan. The Taiwan National Health Insurance Administration maintains extensive databases that cover approximately 99% of the Taiwanese population. We accessed the Longitudinal Health Insurance Database of Patients with Catastrophic Illnesses, and National Health Insurance Research Database (NHIRD). These databases contain detailed information on diagnoses, treatments, surgical procedures, and patient demographics from 1 January 2000 to 31 December 2020, with follow-up extending to 31 December 2021. All personal identifiers were encrypted or removed prior to analysis to ensure confidentiality. The Research Ethics Committee of the China Medical University and Hospital approved this study and waived the requirement for informed consent (CRREC-109-018, approved on 31 January 2026).

### 2.2. Study Population

**Figure 1:**
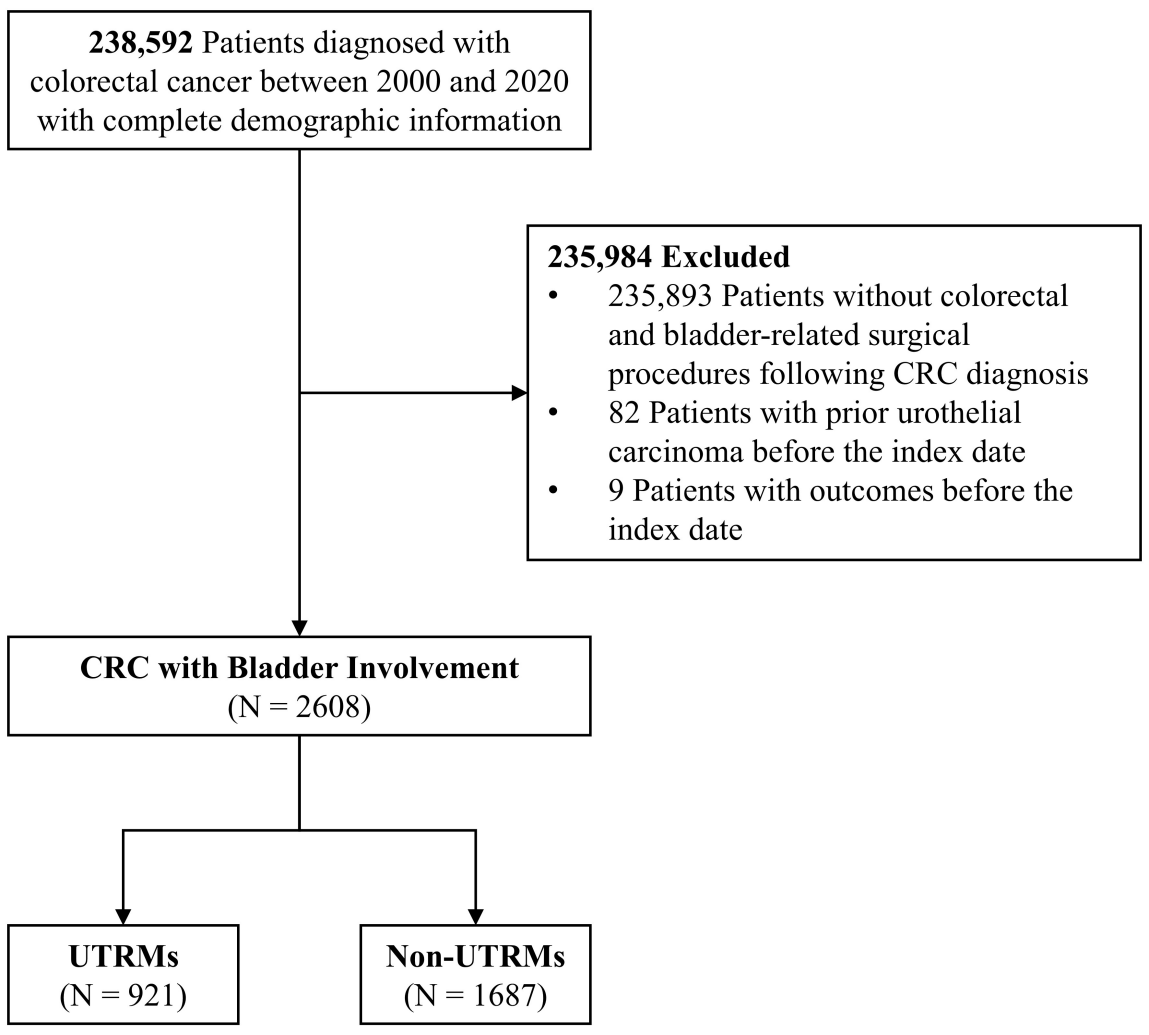
Flowchart of patient selection for the nationwide cohort of colorectal cancer patients with bladder involvement.

The study population included patients newly diagnosed with CRC (International Classification of Diseases, Ninth Revision, Clinical Modification (ICD-9-CM) codes: 153.x, 154.x, and ICD-10-CM: C18-20) who were ≥20 years. All included patients were registered in the catastrophic illness database of the National Health Insurance program of Taiwan, which provides comprehensive coverage and waives copayments for patients with qualifying serious conditions such as cancer. Patients with CRC involving the bladder were operationally identified based on the coexistence of colorectal and bladder-related surgical procedures following a new diagnosis of CRC. Colorectal and bladder-related surgeries were identified using specific surgical procedure codes (Supplementary Table S1). This operational definition was intended to identify patients with clinically advanced CRC who underwent combined colorectal and bladder-related surgical management. Because pathological confirmation of direct bladder invasion was not consistently available in the administrative database, this approach was designed to capture patients with clinically suspected or surgically managed bladder involvement. Patients with a history of urothelial carcinoma (ICD-9-CM codes: 188.x, 189.x; ICD-10-CM: C65–C68) before CRC diagnosis were excluded. The detailed study protocol is shown in Figure 1.

### 2.3. Definitions of Urinary Tract Related Manifestations and Covariates

Patients with CRC involving the bladder were classified according to the presence or absence of prediagnostic urinary tract-related manifestations (UTRMs). UTRMs were operationally defined as urinary tract-related manifestations occurring within 6 months before the index date of CRC diagnosis. This prediagnostic window was selected to capture clinically relevant urinary manifestations occurring close to CRC diagnosis while reducing potential misclassification from unrelated benign urological conditions and minimizing reverse causation. The UTRMs cohort comprised patients with at least two outpatient or inpatient diagnoses related to urinary tract-related manifestations, accompanied by corresponding medication prescriptions (Table S1), during the 6-month period preceding CRC diagnosis. UTRMs included diagnostic codes related to urinary frequency, urgency, dysuria, hematuria, and urinary tract infection (UTI) recorded in the National Health Insurance Research Database. Although UTI represents a clinical diagnosis rather than an isolated symptom, UTI-related diagnostic codes were included to capture clinically relevant urinary tract presentations encountered in routine clinical practice. For patients with UTI-related diagnoses, antibiotic prescriptions recorded during the 6-month prediagnostic period were used as an operational marker of clinically treated infections and were not analyzed as therapeutic exposures.

Patients in the non-UTRMs cohort were defined as those with at most one UTRM-related diagnosis and no more than one corresponding medication prescription during the same 6-month prediagnostic period. The index date was uniformly defined as the date of initial CRC diagnosis for all patients.

Baseline comorbidities were identified using administrative claims records prior to CRC diagnosis. A comorbidity was considered present if the corresponding diagnosis was recorded in at least three outpatient claims or at least one inpatient admission during the prediagnostic period. The assessed comorbidities included hypertension, hyperlipidemia, diabetes mellitus, benign prostatic hyperplasia, chronic kidney disease, cardiovascular diseases, and cerebrovascular diseases, as defined by the International Classification of Diseases, Ninth and Tenth Revisions (ICD-9-CM and ICD-10-CM). To reduce potential misclassification, benign prostatic hyperplasia was required to be diagnosed more than 6 months before CRC diagnosis. Detailed ICD codes used for comorbidity identification are provided in Supplementary Table S1.

### 2.4. Outcomes

The primary outcomes of this study were CRC-specific mortality and cancer recurrence. Recurrence events, including local recurrence, regional lymph node recurrence, and distant metastasis, were ascertained using data from the Taiwan Cancer Registry Database [16]. CRC-specific mortality was determined based on records from the national death registry within the National Health Insurance database.

All patients were followed from the index date until the occurrence of death or the end of the study period, whichever occurred first. Because death may preclude the observation of recurrence, cause-specific hazard models were used to evaluate the association between UTRMs and recurrence while treating death as a competing event.

### 2.5. Statistical Analysis

Descriptive statistics were used to summarize baseline characteristics, presenting categorical variables as counts with percentages and continuous variables such as age as means with standard deviations. Between-group differences for patients with and without UTRMs were examined using Wilcoxon rank sum test for continuous variables and Chi-square analyses for categorical variables, including demographics, comorbidities, and medication usage. Variables included in the propensity score model comprised age, sex, occupation, income level, hypertension, hyperlipidemia, diabetes mellitus, chronic kidney disease, coronary artery disease, cerebrovascular disease, benign prostatic hyperplasia, and medication usage.

To mitigate selection bias and confounding effects, we implemented the inverse probability of treatment weighting (IPTW) methodology [17]. This approach generates propensity scores (PS) by modeling the likelihood of UTRMs diagnosis based on comprehensive baseline characteristics. The weighting scheme assigned values of 1/PS to UTRMs patients and 1/(1−PS) to non-UTRMs patients, effectively creating a pseudo-population in which the covariate distributions were independent of the UTRMs status. Importantly, weight stabilization incorporates marginal UTRMs probabilities in the numerator to reduce extreme weight values [17]. Covariate balance achievement was verified using standardized mean differences, with values below 0.10 indicating a successful balance [18,19]. Moreover, incidence rates of CRC-specific mortality and recurrence were calculated per 1000 person-years by dividing the number of events by the total person-time at risk. IPTW-weighted cumulative event probability curves were constructed to visualize recurrence and CRC-specific mortality outcomes, and differences between groups were assessed using IPTW-adjusted log-rank tests. IPTW-weighted Cox proportional hazard models were used to estimate hazard ratios (HRs) and 95% confidence intervals (CIs) with adjustments for potential confounders, including age, sex, comorbidities, and medication usage. Subgroup analyses were performed for exploratory purposes to evaluate the consistency of associations across predefined strata.

All statistical analyses were performed using the SAS software (version 9.4; SAS Institute Inc., Cary, NC, USA). Statistical significance was defined as a two-sided *p*-value of <0.05.

## 3. Results

### 3.1. Patient Characteristics

A total of 2608 patients with CRC involving the bladder were included in this study, of whom 921 (35.3%) had prediagnostic UTRMs and 1687 (64.7%) did not. Before IPTW, patients with UTRMs demonstrated different baseline clinical characteristics compared with those without UTRMs. Specifically, patients in the UTRMs group were older (mean age, 65.35 vs. 63.08 years; *p* < 0.0001) and were more likely to be male (74.92% vs. 70.72%; *p* = 0.0221). They also had a greater burden of baseline comorbidities, including hypertension, diabetes mellitus, hyperlipidemia, cerebrovascular disease, and benign prostatic hyperplasia. Occupation and income level were generally comparable between the two groups.

**Figure 2:**
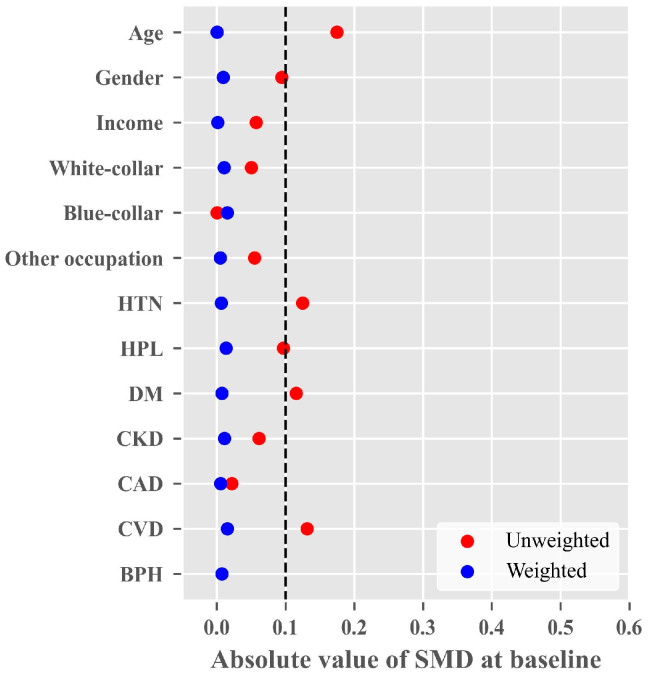
Absolute Standardized Mean Differences Before and After IPTW Adjustment.

Before weighting, several baseline characteristics showed meaningful imbalance between the UTRMs and non-UTRMs groups, as indicated by standardized mean differences (SMDs) greater than 0.1 (Table 1). After IPTW adjustment, all measured covariates achieved excellent balance, with absolute SMD values below 0.1 and most below 0.02, indicating substantial improvement in comparability between the two groups (Figure 2). Detailed SMDs for all baseline characteristics and comorbidities before and after IPTW weighting were listed in Supplementary Table S2.

### 3.2. Association of Prediagnostic UTRMs with Recurrence in CRC with Bladder Involvement

#### 3.2.1. Recurrence Outcomes

**Figure 3:**
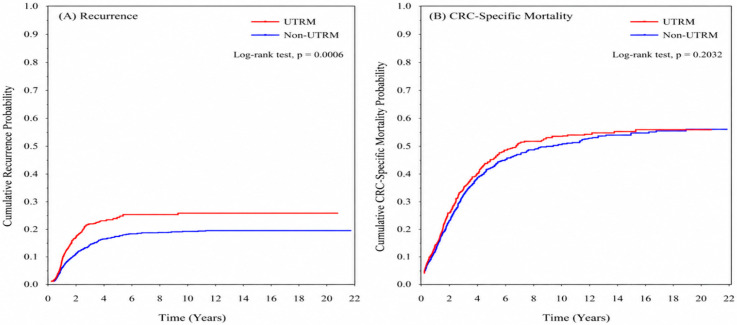
IPTW-weighted cumulative event probability curves for recurrence (**A**) and CRC-specific mortality (**B**) among patients with colorectal cancer involving the bladder according to prediagnostic UTRMs status. Y-axis represents cumulative incident probability on a scale of 0 to 1.

The IPTW-weighted cumulative event probability curves for disease recurrence are shown in Figure 3A and demonstrated a significantly higher recurrence risk among patients with UTRMs compared with those without UTRMs (log-rank test, *p* < 0.001). Consistent with these findings, patients with UTRMs had a significantly increased risk of recurrence (crude HR, 1.58; 95% CI, 1.27–1.95), which remained significant after multivariable adjustment (adjusted HR, 1.57; 95% CI, 1.26–1.95) (Table 2). Patients with UTRMs who required antibiotic treatment also had an increased risk of recurrence (adjusted HR, 4.17; 95% CI, 1.90–9.15). However, this finding should be interpreted cautiously given the limited sample size of this subgroup.

#### 3.2.2. Cancer-Specific Mortality

During the study period, 388 deaths occurred in the UTRMs group and 730 deaths occurred in the non-UTRMs group (Table 2). The IPTW-weighted curves for CRC-specific mortality did not differ significantly between the two groups (log-rank test, *p* = 0.203) (Figure 3B). Although the incidence rate of CRC-specific mortality was numerically higher among patients with UTRMs (85.99 vs. 73.85 per 1000 person-years), neither the crude nor adjusted hazard ratios demonstrated a statistically significant association between UTRMs and CRC-specific mortality (adjusted HR, 1.13; 95% CI, 0.98–1.29).

Among patients with UTRMs, those with UTI-related manifestations requiring antibiotic treatment had a higher observed risk of CRC-specific mortality (adjusted HR, 2.18; 95% CI, 1.25–3.81). However, given the small number of patients in this subgroup (*n* = 32), this finding should be considered exploratory and interpreted with caution.

### 3.3. Subgroup Analyses

Supplementary Table S3 summarizes the crude recurrence and CRC-specific mortality rates stratified by age group and sex. Recurrence rates were generally higher among patients with UTRMs than among those without UTRMs, particularly among middle-aged and older patients (40–59 years: 18.80% vs. 15.57%; ≥60 years: 17.62% vs. 12.29%) and in male patients (18.26% vs. 13.75%), whereas the difference was less pronounced in female patients (17.75% vs. 14.98%). In contrast, CRC-specific mortality rates were broadly comparable between groups across age and sex strata.

We further explored the association between UTRMs and oncologic outcomes across predefined subgroups stratified by demographic characteristics and comorbidities (Supplementary Table S4). Overall, the association between UTRMs and disease recurrence remained generally consistent across most clinically relevant strata, including age, sex, socioeconomic status, and comorbidity profiles. Although the magnitude of the hazard ratios varied across subgroups, the overall direction of association remained similar. In contrast, associations between UTRMs and CRC-specific mortality were less consistent across subgroups and should be interpreted cautiously. Given the exploratory nature of these analyses and the limited sample size in certain strata, these findings should be considered hypothesis-generating insights rather than establishing definitive effect modification.

## 4. Discussion

This study represents a large-scale population-based investigation of UTRMs in patients with colorectal cancer involving the bladder. Using nationwide administrative and cancer registry data, we observed a significant association between prediagnostic UTRMs and disease recurrence in this high-risk population. These findings suggest that urinary tract-related manifestations, which often overlap with common benign urological conditions in routine clinical practice, may provide clinically relevant information regarding recurrence risk in patients with locally advanced CRC involving the bladder.

In the present study, bladder involvement in locally advanced CRC was operationally identified using bladder-related surgical procedure codes, including partial or radical cystectomy. Because pathological confirmation of bladder invasion was not consistently available, our definition was intended to capture patients with clinically suspected or surgically managed bladder involvement. Previous studies have demonstrated that true tumor invasion cannot always be reliably distinguished from inflammatory adhesions before or during surgery; therefore, en bloc resection is generally recommended to achieve adequate oncologic clearance and avoid tumor violation [4,6,7,9]. Accordingly, patients undergoing surgery for suspected bladder involvement may represent a clinically heterogeneous population that includes both pathological bladder invasion and inflammatory adhesions [6,7,10,11].

Importantly, pathological bladder invasion and positive surgical margins have been associated with increased risks of recurrence and distant metastasis in previous studies [9,10,11]. Luo et al. reported that patients with preoperative colovesical fistulas experienced substantially higher recurrence rates than those with inflammatory adhesions (20.6% vs. 6%), suggesting that clinically significant bladder involvement may be associated with adverse oncologic outcomes [10]. Although pathological invasion, fistula formation, and surgical margin status were not available in the present database, these observations provide indirect support for our findings, as UTRMs may represent clinically apparent manifestations of more extensive local disease or clinically significant bladder involvement.

Building on these observations, our IPTW-weighted analyses demonstrated that patients with UTRMs had a significantly higher risk of disease recurrence, with an adjusted hazard ratio of 1.57. Patients with UTRMs who required antibiotic treatment also showed a higher recurrence risk, although this finding should be interpreted cautiously given the limited sample size of this subgroup. In our cohort, approximately one-third of patients with CRC involving the bladder presented with UTRMs, a proportion broadly consistent with previous surgical series reporting urinary symptoms and urinary tract infections among patients with bladder involvement [8,10,11].

Several studies have reported discrepancies between clinically suspected and pathologically confirmed bladder invasion, suggesting that preoperative clinical manifestations do not always accurately reflect the extent of local disease [9,11,12]. Nevertheless, previous surgical series of CRC involving the bladder have reported recurrence rates ranging from approximately 10% to 46% [8,9,10,11,12], and 5-year overall ranging from 62% to 73% following surgical treatment [6,7]. Despite differences in patient selection and follow-up duration, our findings suggest that UTRMs may serve as readily identifiable preoperative manifestations associated with an increased risk of disease recurrence in patients with locally advanced CRC involving the bladder.

Although UTRMs were significantly associated with disease recurrence, only a non-significant trend toward increased CRC-specific mortality was observed in the overall cohort. This discrepancy may reflect the multifactorial nature of long-term survival in advanced CRC, which is influenced by subsequent treatments, surveillance intensity, and competing clinical factors. Advances in surgical techniques [20,21,22] and systemic therapies [4,23,24,25] may partly attenuate the impact of local disease-related factors at presentation on CRC-specific mortality, even among patients at higher risk of recurrence. In contrast, recurrence may represent an earlier oncologic event that is more directly influenced by local disease characteristics, which could explain the stronger association observed between UTRMs and recurrence than with CRC-specific mortality. Furthermore, long-term outcomes in advanced CRC are substantially influenced by multidisciplinary treatment strategies that could not be fully captured in the present database. Therefore, residual treatment-related confounding cannot be excluded when interpreting the mortality findings.

In subgroup analyses, the association between prediagnostic UTRMs and disease recurrence appeared generally consistent across clinically relevant strata, including age, sex, socioeconomic status, and comorbidity profiles. In contrast, associations with CRC-specific mortality were less consistent across subgroups. Given the exploratory nature of these analyses and the small numbers in some subgroups, these findings should be interpreted with caution and require further validation. Future studies incorporating prospective symptoms assessment and more detailed clinical and pathological data are warranted to further clarify the relationship between UTRMs and oncologic outcomes.

From a clinical perspective, these findings highlight the potential relevance of prediagnostic UTRMs as readily obtainable clinical manifestations in patients with suspected locally advanced CRC involving the bladder. Although UTRMs should not be used in isolation to determine surgical strategy, their presence may identify patients at increased risk of recurrence. Therefore, patients presenting with UTRMs may warrant careful preoperative evaluation, multidisciplinary treatment planning, and closer postoperative surveillance.

This study has several limitations. First, the retrospective design precludes causal inference and may be subject to misclassification bias inherent to the use of administrative claims data. Second, bladder involvement was operationally identified using surgical procedure codes and may not fully correspond to pathological confirmation of direct bladder invasion, potentially introducing heterogeneity in disease extent and uncertainty in distinguishing true tumor invasion from inflammatory adhesions or clinically suspected bladder involvement. Third, the absence of detailed pathological characteristics, such as depth of bladder invasion, lymphovascular and perineural invasion and surgical margin status, as well as limited treatment-related information regarding treatment intent, sequencing, completion, and response, precluded a more detailed evaluation of treatment effects and disease progression. Additionally, unmeasured confounding factors, including symptom severity, functional status, and laboratory or imaging findings, could not be fully accounted for. These unavailable clinical factors may have contributed to residual confounding. Therefore, the observed associations should be interpreted as prognostic rather than causal. Furthermore, the small number of patients in the antibiotic-treated subgroup (*n* = 32) limits the interpretation of these findings. In addition, differences in healthcare systems, referral patterns, and treatment strategies may limit the generalizability of these findings to other populations or healthcare settings. Finally, UTRMs were identified using diagnostic coding algorithms rather than prospective symptom assessment. Consequently, some manifestations may have reflected coexisting benign urological conditions rather than direct tumor-related effects. Furthermore, the inclusion of both symptom-based manifestations and UTI-related diagnoses in the operational definition of UTRMs may have introduced heterogeneity in exposure classification, potentially influencing the observed associations. Nevertheless, the nationwide population-based design, large sample size, and longitudinal follow-up provide valuable real-world evidence regarding the association between prediagnostic UTRMs and oncologic outcomes in patients with CRC involving the bladder.

Future studies incorporating prospective symptom assessment, pathological validation, and more detailed clinical and treatment-related information are warranted to further clarify the association between UTRMs and oncologic outcomes and to refine management strategies in this high-risk patient population.

## 5. Conclusions

This population-based study demonstrated that prediagnostic UTRMs were significantly associated with an increased risk of recurrence in patients with locally advanced colorectal cancer involving the bladder. These findings suggest that UTRMs may serve as readily obtainable preoperative clinical manifestations associated with recurrence risk and may support more comprehensive preoperative evaluation and postoperative surveillance in this high-risk population. Further prospective studies incorporating detailed pathological and treatment-related data are warranted to better define the prognostic significance and clinical implications of these associations.

## Figures and Tables

**Table 1:** Baseline Characteristics of Patients with CRC Involving the Bladder Before IPTW Adjustment.

	Overall	UTRMs	Non-UTRMs	
	(*n* = 2608)	(*n* = 921)	(*n* = 1687)	SMD
Age, Mean ± SD	63.88 ± 13.05	65.35 ± 12.92	63.08 ± 13.05	0.17
Gender				
Male	1883 (72.20%)	690 (74.92%)	1193 (70.72%)	0.09
Female	725 (27.80%)	231 (25.08%)	494 (29.28%)	
Occupation				
White collar	907 (34.78%)	306 (33.22%)	601 (35.63%)	0.05
Blue collar	1017 (39.00%)	359 (38.98%)	658 (39.00%)	0.00
Others	684 (26.23%)	256 (27.80%)	428 (25.37%)	−0.05
Monthly income, NTD	22,899.60 ± 20,040.00	22,158.40 ± 19,514.00	23,304.30 ± 20,316.00	−0.06
Comorbidities				
HTN	1195 (45.82%)	459 (49.84%)	736 (43.63%)	−0.12
HPL	407 (15.61%)	165 (17.92%)	242 (14.34%)	−0.10
DM	476 (18.25%)	195 (21.17%)	281 (16.66%)	−0.12
CKD	68 (2.61%)	30 (3.26%)	38 (2.25%)	−0.06
CAD	269 (10.31%)	99 (10.75%)	170 (10.08%)	−0.02
CVD	416 (15.95%)	176 (19.11%)	240 (14.23%)	−0.13
BPH	359 (13.77%)	273 (29.64%)	86 (5.10%)	−0.68

HTN: hypertension; HPL: hyperlipidemia; DM: diabetes mellitus; CKD: chronic kidney disease; CAD: coronary artery disease; CVD: cerebral vascular disease; BPH: benign prostate hyperplasia. CRC: colorectal cancer; IPTW: inverse probability of treatment weighting; NTD: New Taiwan Dollar.

**Table 2:** Risk of CRC-specific mortality and recurrence according to prediagnostic UTRMs status in patients with CRC involving the bladder.

	Event	Person Year	Incidence Rate	Crude HR (95% CI)	Adjusted Model HR (95% CI)
Death					
Without UTRMs (*n* = 1687)	730	9885.15	73.85	REF	REF
With UTRMs (*n* = 921)	388	4512.40	85.99	1.12 (0.98–1.29)	1.13 (0.98–1.29)
Within 1–90 Days Prior to Index (*n* = 506)	209	2475.25	84.44	1.13 (0.96–1.33)	1.16 (0.98–1.36)
Within 91–180 Days Prior to Index (*n* = 415)	179	2037.15	87.87	1.12 (0.93–1.34)	1.09 (0.90–1.32)
Without Antibiotic Treatment (*n* = 889)	373	4411.29	84.56	1.11 (0.97–1.27)	1.11 (0.97–1.27)
With Antibiotic Treatment (*n* = 32)	15	101.11	148.35	1.89 (1.09–3.30) *	2.18 (1.25–3.81) **
Recurrence					
Without UTRMs (*n* = 1687)	238	9236.13	25.77	REF	REF
With UTRMs (*n* = 921)	167	4102.01	40.71	1.58 (1.27–1.95) ***	1.57 (1.26–1.95) ***
Within 1–90 Days Prior to Index (*n* = 506)	95	2239.84	42.41	1.67 (1.29–2.14) ***	1.66 (1.30–2.14) ***
Within 91–180 Days Prior to Index (*n* = 415)	72	1862.17	38.66	1.45 (1.08–1.93) *	1.43 (1.05–1.95) *
Without Antibiotic Treatment (*n* = 889)	160	4025.85	39.74	1.52 (1.23–1.89) ***	1.52 (1.22–1.90) ***
With Antibiotic Treatment (*n* = 32)	7	76.16	91.92	4.49 (2.12–9.52) ***	4.17 (1.90–9.15) ***

Incidence rate is equal to the number of events per 1000 person-year. Adjusted models adjusted age, gender, occupation, income, and comorbidities (hypertension, hyperlipidemia, diabetes mellitus, chronic kidney disease, coronary artery disease, cerebral vascular disease and benign prostate hyperplasia) * 0.01< *p* value < 0.05; ** 0.001 < *p* value < 0.01; *** *p* value < 0.001. CRC: colorectal cancer; UTRMs: urinary tract–related manifestations; HR: hazard ratios; CI: confidence intervals; REF: reference group.

## Data Availability

Data are available from the National Health Insurance Research Database (NHIRD) published by the Taiwan Ministry of Health and Welfare. Due to legal restrictions imposed by the government of Taiwan in relation to the “Personal Information Protection Act”, data cannot be made publicly available. Requests for data can be sent as a formal proposal to the Health and Welfare Data Science Center, Taiwan.

## Supplementary Materials

The following supporting information can be downloaded at: https://www.mdpi.com/article/10.3390/jcm15155867/s1, Table S1: Classification of Diseases and Medications Under Study Based on the International Statistical Classification of Diseases (ICD-9 and ICD-10), Order Code and Drug Code; Table S2: Standardized mean differences (SMDs) in baseline characteristics and comorbidities before and after IPTW weighting; Table S3: Recurrence and CRC-Specific Mortality by Age and Sex; Table S4: Subgroup Analyses of the Association Between UTRMs and Oncologic Outcomes According to Baseline Characteristics and Comorbidities.

## Abbreviations

CRC	Colorectal cancer
UTRMs	Urinary tract related manifestations
BPH	benign prostatic hyperplasia
CKD	chronic kidney disease
CAD	coronary artery disease
IPTW	inverse probability of treatment weighting
