# Supplementary material for: Prediagnostic Urinary Tract-Related Manifestations and Recurrence Risk in Colorectal Cancer with Bladder Involvement
Source: J Clin Med. 2026 Jul 27;15(15):5867. doi: 10.3390/jcm15155867 (PMC13467500; doi:10.3390/jcm15155867)
Supplement: Supplementary file 1 [file jcm-15-05867-s001.zip › jcm-4304300-supplementary.pdf]

Table S1 Classification of Diseases and Medications Under Study Based on the International Statistical Classification of Diseases (ICD-9 and ICD-10), Order Code and Drug Code

Name	Code
Disease	
Colorectal carcinoma	153, 154, C18, C19, C20, C21
Urothelial carcinoma	188, 189, C64, C65, C66, C67, C68
Hypertension	401, 402, 403, 404, 405, I10, I11, I12, I13, I15
Hyperlipidemia	272, E75, E77, E78
Diabetes Mellitus	250, E11
Chronic Kidney Disease	249.40, 403.01, 403.11, 403.91, 585, E08.22, E09.22, E10.22, E11.22, E13.22, I12.0, I12.9, I13.10, I13.11, N18
Cerebral Vascular Disease	430, 431, 432, 433, 434, 435, 436, 437, I60, I61, I62, I63, I66, I67, I68
Coronary Artery Disease	410, 411, 412, 413, 414, I20, I21, I22, I23, I24
Benign Prostatic Hyperplasia	600.0, N40.0, N40.1
Urinary Tract Related Manifestations	595, 596.5, 596.6, 596.7, 596.8, 596.9, 597, 598.01, 599.0, 599.5, 599.7, 599.83, 599.84, 599.89, 599.9, 600.0, 788, N23, N30, N32.8, N32.9, N34, N36.8, N36.9, N37, N39, N40.0, N40.1, R30, R31, R32, R33, R34, R35, R36, R39
Surgery	
Colectomy	73011B, 73012B, 73013B, 73014B, 73017B, 73046B, 73047B, 73048B, 74205B, 74216B, 74220B, 74222B, 74223B, 73047B, 73048B, 74213B, 74214B
Cystectomy or Endoscopic surgery	78008C, 78009B, 78010C, 78011B, 78012B, 78013B, 78014B, 78015B, 78016B, 78018B, 78021B, 78039B, 78040B, 78041B, 78042B, 78043B, 78044B, 78045B, 78046B, 79407C
Medications	
Antibiotics for urinary tract infection	A029623100, A030871100, A030874100, A032295100, A035519100, AB33540100, AC29599100, AC31072100, AC310721G0, AC31073100, AC32324100, AC34037100, AC340371G0, AC34038100, AC35844100, AC36693100, AC37125100, AC37390100, AC38353100, AC383531G0, AC43928100, AC59758100, AC19899100, A007786100, A008743100, A009632100, A010576100, A012147100, A015345100, A019577100, A026073100, A0431141G0, AC04514100, AC04919100, AC049191G0, AC07936100, AC08662100, AC08855100, AC09256100, AC10120100, AC10282100, AC102821G0, AC10764100, AC11562100, AC115621G0, AC12763100, AC12951100, AC13366100, AC15616100, AC16020100, AC16162100, AC17433100, AC19649100, AC24362100, AC24592100, AC24964100, AC249641G0, AC25555100, AC255551G0, AC26205100, AC32535100, AC33163100, AC36083100, AC43114100, AC55910100, AC559101G0, AC45699100, AC48892100, AC59292100, A039271100, A039488100, A039722100, AB58253100, AC36580100, AC38089100, AC38090100, AC38368100, AC38528100, AC38747100, AC39170100, AC40429100, AC45201100, AC47993100, AC49619100, BB17692100, BB21062100, AB47516100, AB49696100, AB52615100, AB56626100, AB57839100, AC46634100, AC49452100, AC50104100, AC51715100, AC57101100, AC57242100, AC57300100, AC58044100, AC59888100, BB25828100, BC26772100, BC26837100,

	AC48694100<br>BC19086100, A001850100, A001866100, A002445100, A010382100, A013093100, A016099100, A034909100, AC00373100, AC02444100, AC06821100, AC09609100, AC096091G0, AC10415100, AC16378100, AC17859100, AC18973100, AC19135100, AC20405100, AC25079100, N007897100, N011585100, NC03419100, NC034191G0
Medications for lower urinary tract symptoms	AC27361100, AC30803100, AC34366100, AC35009100, AC35069100, AC35278100, AC35467100, AC36280100, AC36370100, AC38056100, AC40424100, AC41360100, AC41944100, BC24437100, BC22783100, BC22784100, BC23568100, BC26216100, BC26217100
Medications for hematuria	A007976100, A022905100, A026041100, A0270441G0, A031110100, A031833100, AB41226100, AB412261G0, AC23220100, AC24282100, AC24350100, AC25316100, AC253161G0, AC25460100, AC25716100, AC26026100, AC27044100, AC27163100, AC31006100, AC32579100, AC36132100, AC41069100, AC410691G0, AC43941100, AC50057100, NC15392100, AC24754100, AC25842100, AC26093100, AC26303100, AC26330100, AC263301G0, AC33892100, AC57907100

Table S2 Standardized mean differences (SMDs) in baseline characteristics and comorbidities before and after IPTW weighting

	Case (UTRMs) (N = 921)	Control (non-UTRMs) (N = 1,687)	Before IPTW SMD	After IPTW SMD
Age, Mean ± SD	65.35 ± 12.92	63.08 ± 13.05	0.17481	-0.00053
Gender			0.09454	-0.00944
Male	690 (74.92%)	1,193 (70.72%)		
Female	231 (25.08%)	494 (29.28%)		
Occupation				
White collar	306 (33.22%)	601 (35.63%)	0.05054	0.01075
Blue collar	359 (38.98%)	658 (39.00%)	0.00051	-0.01532
Others	256 (27.80%)	428 (25.37%)	-0.05492	0.00536
Monthly income, NTD	22,158.40 ± 19,514.00	23,304.30 ± 20,316.00	-0.05753	-0.00131
Comorbidities				
HTN	459 (49.84%)	736 (43.63%)	-0.1247	0.00636
HPL	165 (17.92%)	242 (14.34%)	-0.09718	0.01347
DM	195 (21.17%)	281 (16.66%)	-0.1155	0.00734
CKD	30 (3.26%)	38 (2.25%)	-0.06142	0.01145
CAD	99 (10.75%)	170 (10.08%)	-0.02201	-0.00579
CVD	176 (19.11%)	240 (14.23%)	-0.13131	0.01559
BPH	273 (29.64%)	86 (5.10%)	-0.68478	0.00741

HTN: hypertension; HPL: hyperlipidemia; DM: diabetes mellitus; CKD: chronic kidney disease; CAD: coronary artery disease; CVD: cerebral vascular disease; BPH: benign prostate hyperplasia.

Table S3 Recurrence and CRC-Specific Mortality by Age and Sex

	Recurrence						CRC-specific mortality					
	URTMs			Non-UTRMs			URTMs			Non-UTRMs		
	N	Event	Rate	N	Event	Rate	N	Event	Rate	N	Event	Rate
Age												
20-39	25	6	24.00%	76	21	27.63%	25	11	44.00%	76	39	51.32%
40-59	266	50	18.80%	578	90	15.57%	266	86	32.33%	578	223	38.58%
60+	630	111	17.62%	1,033	127	12.29%	630	291	46.19%	1,033	468	45.30%
Gender												
Male	690	126	18.26%	1,193	164	13.75%	690	289	41.88%	1,193	515	43.17%
Female	231	41	17.75%	494	74	14.98%	231	99	42.86%	494	215	43.52%

Table S4 Subgroup Analyses of the Association Between UTRMs and Oncologic Outcomes According to Baseline Characteristics and Comorbidities

	CRC-specific mortality		Recurrence	
	HR (95% CI)	p value	HR (95% CI)	p value
Age				
20 to 39	0.95 (0.44 - 2.04)	0.8947	0.86 (0.33 - 2.28)	0.768
40 to 59	1.08 (0.82 - 1.41)	0.5937	1.69 (1.15 - 2.48)	0.0074
60+	1.17 (0.99 - 1.38)	0.0632	1.73 (1.29 - 2.32)	0.0003
Gender				
Male	1.20 (1.02 - 1.41)	0.0315	1.71 (1.31 - 2.24)	0.0001
Female	0.97 (0.76 - 1.24)	0.7972	1.22 (0.82 - 1.81)	0.3325
Occupation				
White collar	1.11 (0.88 - 1.41)	0.379	1.65 (1.15 - 2.36)	0.0066
Blue collar	1.11 (0.90 - 1.37)	0.3212	1.48 (1.06 - 2.07)	0.0212
Others	1.16 (0.89 - 1.51)	0.2774	1.56 (1.00 - 2.43)	0.0524
Income				
≤ 15000	1.20 (0.92 - 1.56)	0.1806	1.66 (1.08 - 2.58)	0.0224
15000–20000	1.08 (0.88 - 1.32)	0.4856	1.51 (1.07 - 2.14)	0.0198
> 20000	1.17 (0.90 - 1.52)	0.2335	1.57 (1.06 - 2.33)	0.0262
HTN				
No	1.06 (0.87 - 1.29)	0.5893	1.71 (1.27 - 2.30)	0.0004
Yes	1.22 (1.01 - 1.48)	0.0445	1.41 (1.01 - 1.97)	0.0429
HPL				
No	1.13 (0.97 - 1.31)	0.109	1.56 (1.23 - 1.98)	0.0003
Yes	1.13 (0.79 - 1.61)	0.5178	1.56 (0.89 - 2.73)	0.1186
DM				
No	1.07 (0.92 - 1.25)	0.3837	1.46 (1.14 - 1.88)	0.0029
Yes	1.33 (0.97 - 1.82)	0.0743	1.93 (1.12 - 3.35)	0.0189
CKD				
No	1.11 (0.96 - 1.27)	0.1538	1.57 (1.26 - 1.96)	<.0001
Yes	0.94 (0.32 - 2.79)	0.9072	0.12 (0.01 - 1.36)	0.0872
CAD				
No	1.07 (0.93 - 1.24)	0.363	1.48 (1.18 - 1.87)	0.0009
Yes	1.82 (1.21 - 2.76)	0.0043	3.02 (1.43 - 6.37)	0.0037
CVD				
No	1.12 (0.97 - 1.30)	0.134	1.58 (1.24 - 2.00)	0.0002
Yes	1.13 (0.82 - 1.57)	0.4512	1.56 (0.83 - 2.91)	0.1657
BPH				
No	1.16 (1.01 - 1.34)	0.0429	1.61 (1.28 - 2.01)	<.0001
Yes	0.96 (0.64 - 1.43)	0.8263	1.09 (0.50 - 2.35)	0.8293

HTN: hypertension; HPL: hyperlipidemia; DM: diabetes mellitus; CKD: chronic kidney disease; CAD: coronary artery disease; CVD: cerebral vascular disease; BPH: benign prostatic hyperplasia.
Hazard ratios were adjusted for age, sex, occupation, income level, hypertension, hyperlipidemia, diabetes mellitus, chronic kidney disease, coronary artery disease, cerebrovascular disease, and benign prostatic hyperplasia.
